# Anterior Poliomyelitis

**Published:** 1916-09

**Authors:** 


### Anterior Poliomyelitis

We know no more about the therapeutics, prophylaxis and etiology of this disease than we did thirty years ago, except in the sense that what little we do know is more generally diffused among the profession and the laity and that certain items are better supported by scientific study, as for instance, that the nasal secretion, washings from the rectum, blood and tissues are infectious to monkeys and rabbits, by way of mucous membrane, even the abraded skin, subcutaneous injection and even by stomach in massive doses. It is possible, too, that claims of discovery of a specific bacterium, of a serum or vaccine, of certain therapeutic measures not directly connected with the potential bacterial cause, etc., may be confirmed. (See Flexner's article Archives of Pediatrics, August).

So far as statistics can be relied upon and properly compared, and with due allowance for fluctuations in incidence from year to year, it may be doubted whether poliomyelitis is more prevalent than formerly. We are, officially and privately, waking up to our duty to discover what the disease really is and to check it in some way.

In many respects, poliomyelitis is a puzzle. Not only are we ignorant of its true nature but it does not correspond to the general principles learned from knowledge of or empiric experience with other diseases. It has the characteristics of an infection but, except experimentally, it violates most of the rules that may be applied to infections. It is almost absolutely limited to young children, approximately to those under 5. Most ''children's diseases'' are merely human beings' diseases. Children succumb because they are not immunized. If they happen to escape, there remains a liability to infection, at any time of life, subject to greater general resistance, lack of exposure, or what may be reasonably explained as immunity by heredity or by an abortive attack. Small pox, for example, is just as much a children's disease as scarlet fever except that by the introduction of artificial methods of prophylaxis, children of ambulant age are better protected than adults. A few local epidemics of poliomyelitis have attacked over 1% of the total population but the average alarming epidemic does not attack even 1 per mille. Remembering that children under 6 comprise about 11% of the total population, these statistics mean that an epidemic occurring in virgin soil attacks only about 10% of the particularly susceptible material and that the mere element of advancing age rapidly produces an effective resistance, which is almost absolute for adults, even if not previously immunized by an attack. For an ordinary epidemic, even of serious degree, 1% or less of young children are attacked.

Direct contagion—remembering that this word means an infection so communicable that not even actual contact is required for its transmission—is seldom noted even on the post hoc propter hoc argument. The disease does not correspond in period of maximum incidence to the school year and, indeed, occurs mainly in children too young for school. It is no more common in poor, crowded and unsanitary districts than under the opposite conditions; indeed, epidemics of maximum proportionate incidence, occur mainly in villages and the open country. Transmission does not necessarily follow routes of travel nor does an introduced case obviously act as a focus for others.

Fomites furnish infectious material under experimental control, but do not usually serve as centers for spontaneous infection, while epidemics abate in spite of a large number of suppositiously infected centers and, as stated, long before the exhaustion of suppositiously susceptible individuals.

Insects have been incriminated on general principles and experimental infection has been transferred by them but the period of epidemics exceeds that in which either house and body insects on the one hand or out-door insects on the other, are liable to particular suspicion. The erratic distribution of the disease in any given epidemic, seems incompatible with a specific, obligate or essential insect carrier. However, it is possible that these apparent inconsistencies with the entomophoric theory may indicate special, temporary distributions of some particular species or even variety of insect.

Human epidemics are stated not to coincide with epizootics that might indicate a correspondence with a lower animal host, possibly afflicted in a different clinical manner.

No connection has been noted, with water, ice, milk, or other food supplies. It has been suggested that the disease might be a deprivation or special food intoxication, independently of or in connection with an infection, but, on the one hand, its greatest incidence occurs when food is, on the whole most abundant and does not correspond to differences in nutrition, either by classes or individuals, while, on the other hand, the wide regional and class distribution of the disease, and the duration of the period of incidence over food seasons, seems to rule out a specific food intoxication. The age of susceptibility, though practically confined within small limits, is wide in regard to the characteristic diets involved.

It has, of course, been suggested, that the microorganism of poliomyelitis is not strictly specific but that the disease is a clinical variant of some other specific infection, intracellularis, pneumococcic, etc. But, on the one hand, no evidence has been adduced that susceptibility to poliomyelitis is negatived by an acquired immunity to any other specific disease and, on the other hand, it would seem that repeated search for the specific microorganism would certainly have yielded one already familiar if it were the cause. Moreover, how can we explain a general conformity to a given clinical type, occurring in epidemics and to an entirely different type at other times?

